# Social desirability bias in qualitative health research

**DOI:** 10.11606/s1518-8787.2022056004164

**Published:** 2022-11-18

**Authors:** José Patrício Bispo

**Affiliations:** I Universidade Federal da Bahia Instituto Multidisciplinar em Saúde Vitória da Conquista BA Brasil Universidade Federal da Bahia. Instituto Multidisciplinar em Saúde. Vitória da Conquista, BA, Brasil

**Keywords:** Qualitative Research, Bias, Social Desirability, Methodology, Public Health Systems Research

## Abstract

The objective of this essay is to discuss the social desirability bias in qualitative health research. The social desirability bias consists of a systematic research error, in which the participant presents answers that are more socially acceptable than their true opinions or behaviors. Qualitative studies are very susceptible to this type of bias, which can lead to distorted conclusions about the studied phenomenon. Initially, I present the theoretical-conceptual aspects of the social desirability bias. I discuss how its occurrence can be intentional or unintentional, with a distinction between the concepts of self-deception and impression management. Then, I discuss the determining factors of this bias from four dimensions: study design; study context; interviewee's characteristic; interviewer's posture. Finally, I present a systematization of six strategies to be used by qualitative researchers for identifying and controlling social desirability bias.

## INTRODUCTION

Biases can exist in health research, in both quantitative and in qualitative research^1^. Although it is not a new topic, the discussion about biases in qualitative research is still timid and demands greater attention and depth from researchers. According to Althubaiti^2^, the problem of bias is still often ignored in practice. For the author, in most cases bias is introduced unintentionally by researchers in a study, which makes it difficult to recognize. Thus, it is a matter of great relevance for the debate on enhancing the consistency of qualitative studies.

Research bias can be defined as the influence of a factor that causes distortions in the results of the study^3^. These are systematic errors that can occur at all stages of research development^4^: in the planning and design of the study; in the collection of data; and in the analysis and interpretation of information. In any of the steps, the existence of this type of error can compromise the rigor and consistency of the findings of the research.

In qualitative research, the discussion about the existence and treatment of biases is controversial and not consensual. Characteristics inherent to the method itself, such as obtaining data through verbal interaction or observation and the interpretive nature of the analysis, are often pointed out as uncontrollable sources of biases that threaten the credibility of the research^5^. According to Roulston and Shelton^6^, this is due to the presumption of positivist and quantitative values over the qualitative method.

In this debate, it is important to set the boundaries of the epistemic aspects of qualitative health research. According to Guba and Lincoln^7^, qualitative research seeks to encompass the historical, cultural and subjective dimension of human phenomena. Therefore, objectivity and neutrality, so reified in the natural sciences, are not a foundation for qualitative studies. Qualitative research has as its epistemological reference the interpretive constructivist paradigm, in which reflexivity and subjectivity are valued as means for interpreting complex social phenomena^7,8^.

Trad^9^ underscores that epistemic vigilance consists in balancing the valorization of subjectivity with the imperative of producing scientific knowledge. Thus, it is necessary to bear in mind the contradictions of informants and to consider how they might be attempting to manipulate information. Several strategies are used to try to mitigate the intervening factors that could inadequately influence the observed realities or the reports of the study participants^10,11^.

The data collection phase is very susceptible to response biases. Response bias is understood as the existence of research errors resulting from intentionally or unintentionally misrepresented statements^12^. In this situation, respondents alter, censor or misrepresent their true opinions, thoughts and beliefs. As a result, the answers to the questions are not representative of how participants actually behave, think or feel^12,13^.

There are several types of response bias. Some examples are^2,12,14^ the memory bias; the acquiescence bias, also known as the “yea-saying” bias; the apathy bias or the habituation bias; and the social desirability bias. The social desirability bias is understood as the tendency of a study participant to present himself or his social context in a way that is socially acceptable, but not fully corresponding to reality^15^.

The existence of this type of bias is motivated by the predisposition of people to deny socially undesirable traits and claim other socially desirable ones. Therefore, it relates to the desire to say things that will make a good impression on the people with whom they are interacting in a given situation^16^.

Given the existence of socially reproved behaviors or violations of laws and norms, obtaining honest and reliable reports is a major challenge in qualitative research. As highlighted, qualitative health studies are very susceptible to the social desirability bias. However, this is a topic that has received little attention in the debate on the methodological aspects of health research.

The objective of this essay is to discuss the social desirability bias in qualitative health research and to present potential control strategies for this type of bias. The text is structured into three sections. In the first part, I present the conceptual discussion and theoretical-methodological reflections on socially desirable responses. In the second section, I address the determining factors of this type of bias, considering the characteristics of a qualitative study. In the third section, I systematize some strategies for identifying and controlling the social desirability bias.

### Social Desirability Bias: Conceptual and Methodological Aspects

The social desirability bias describes the behavior by study participants of describing themselves in positive terms to create socially appropriate images of themselves or certain situations, instead of responding truthfully and accurately^14^. Motivated by a variety of factors, participants tend to overestimate socially acceptable behaviors, attitudes, and traits and underestimate true opinions and behaviors if they are socially undesirable^13,17^.

Responses that disclose deviations from social norms are viewed reproachfully and, therefore, are difficult to obtain in scientific investigations. In this sense, the results obtained by the studies may fail to reveal many of the aspects of the study object. Consequently, the conclusions presented by the authors may be distorted or not adequately express people's behavior, the functioning of health services or the development of public policies.

Reports of behaviors aligned with socially established patterns are seen as capable of avoiding reactions of contempt and are often associated with potential gains for one's good image. Thus, respondents can distort their responses towards the social norm to maintain a socially favorable self-image^17^.

The social desirability bias is related to controversial issues or behaviors that elude legal, cultural and ethical standards established in each society. Krumpal^17^ uses the denomination of sensitive topics to express subjects considered taboo, reveal illegal behavior or express antisocial attitudes. Thus, obtaining reliable information while researching sensitive topics is a challenge for the social sciences in health care. The author presents three dimensions of sensitive topics: (1) intrusion, since certain issues can be perceived as private or personal; (2) fear of disclosure, regarding the respondents’ concerns about potential risks and consequences of disclosing answers outside the research environment; and (3) social desirability, regarding the distortion of answers relative to the social norm to present a socially favorable self-image.

An important issue in this debate is that the social desirability bias can be intentional or unintentional. According to Paulhus^18^, socially appropriate responses can result from two situations: self-deception and impression management. In self-deception, distortions of responses are motivated by inflated personality attributes and high self-esteem^15^, which favors the tendency of respondents to always see themselves in a positive way^12^. Thus, respondents actually believe that a statement about themselves is true, even if the answer is inaccurate^15^. Self-deception responses are motivated by the constant need for social approval, regardless of what is being addressed^18^.

In turn, impression management concerns the intentional act of misrepresenting the truth as a way of making a good impression^15^. In this situation, respondents deliberately and consciously manage a response to present themselves in a positive way, omitting and misrepresenting facts that may generate unfavorable situations^14^.

Distinguishing these two perspectives is of great relevance in social health research since it allows the separation of determinants that can be controlled and interfered with by the researcher. Self-deception situations are less easily controlled and, in most cases, can only be detected^16^. In turn, impression management is triggered by a specific situation or item, which the study participant attempts to hide or misrepresent^17^. As they are influenced by the characteristics of an item, they are more easily identifiable and also enable the researcher to develop strategies to prevent or circumvent this type of bias.

### Determinants of the Social Desirability Bias

Obtaining information in qualitative research involves the actors (interviewer, respondents and potential viewers), the relationship established between them, the social segments or institutions to which they are linked, the environment in which the study was conducted and the sociocultural norms established. Thus, some characteristics of the study, the circumstances in which it is performed and the positions of the actors involved can facilitate the occurrence of biases. The following is a systematization of the determinants of the social desirability bias.

### Study Design

Before conducting any scientific study, the researcher must carefully defined the proposed objectives, the research methods, the techniques to be used in obtaining data and the selection of participants. An important step to avoid the social desirability bias is to analyze the pertinence and coherence between the objectives and the methodological elements to be followed.

The choice of technique for obtaining data can facilitate or hinder biased responses. Two collection techniques in qualitative research are prone to the social desirability bias: interview and focus group.

Interviews are characterized as a conversation with a purpose and are as the most used technique in qualitative fieldwork^19^. Several factors can contribute to respondents not formulating answers truthfully, including the desire to omit socially reproved opinions and behaviors, the willingness to demonstrate mastery of the content, or even the desire to please the interviewer. The dynamic nature of the interview and the possibility of the interviewer to identify traces of deviations in the respondent's speech allow the targeting and use of resources to minimize biased approaches.

A focus group is a qualitative research technique for collecting information through group interactions^20^. It is based on generating information through the interaction between the participants, rather than asking them question individually^20,21^. Thus, one of the main challenges of focus groups is precisely to promote interaction and debate among the participants and make them not interact only with the moderator^21^. Given the peculiarities of focus groups, they can constitute a technique capable of mitigating or potentiating the bias of social desirability. In the interaction between the participants, a process of self-control of the group can develop, with the ability to inhibit opinions and positions that do not correspond to the reality under discussion. On the other hand, a kind of social micro-pact may develop to collectively hide certain behaviors or practices that may be considered inappropriate.

Regarding the study participants, three groups of people are most commonly requested in qualitative health research: users and caregivers; health professionals; and managers. The bias of the participants’ responses is strongly related to how sensitive the topics covered in the studies are and the criteria for selecting participants. For example, users may feel uncomfortable disclosing risky sexual behaviors or embarrassed to report domestic violence situations. The situation may be even more difficult to handle with managers of aspects related to the way resources are managed or, if any, about illegal behaviors, omissions and fraud.

In fact, obtaining honest answers corresponding to the reality being studied is not an easy task. It is not rare for participants, when asked about a particular practice or the functioning of a particular service, to describe an ideal situation or present the parameters of a given policy instead of reporting the daily reality that they experience.

The mechanisms of participant selection can also influence the occurrence of the social desirability bias. There are two basic ways of selecting participants^22,23^: to previously set the number and characteristics of respondents and to select them according to the needs and questions that appear in the course of the study. In both ways, properly selecting participants imbricated and willing to share true opinions and behaviors is a challenge to ensure rigor and to reduce the occurrence of bias. Inadequate selection can affect subsequent stages of fieldwork and data analysis, as well as hindering actions to control the social desirability bias.

Another important aspect related to the design of the study is the proper elaboration of the instruments. The writing of the script can induce the content of the answer. According to Kaminska and Foulsham^13^, the formulation of the question may imply that there is a socially undesirable behavior or attitude, leading people to respond in a biased way. That is, certain words or phrases in the instrument suggest certain types of answers^12^. Also, the order of the questions can generate biases, since the answer given to a question can influence the answers to subsequent questions.

### Study Context

Contextual factors of the field step have great potential to generate biases. Two main contextual factors influence the occurrence of the social desirability bias: the bystander effect and data confidentiality.

The bystander effect is the presence of one or more people, in addition to the researcher and the participant, at the time of data collection. Given the high probability of negative repercussions, in the presence of a third party the respondent will report fewer socially undesirable responses^17^. In qualitative health research, it is not uncommon for researchers to find themselves in contexts with a third person at the time of data collection. For example, interviews conducted in the users’ homes are almost always followed by other residents, which can be a difficult situation control and may negatively affect the quality of the information provided. The bystander effect can also occur indirectly, as a result of lack of privacy in the research environment. In certain environments, such as health facilities or administrative spaces, where speech can be heard by people in other spaces, there is a greater propensity for distortion of responses.

Regarding the confidentiality of data, it is necessary to assure respondents and make sure that they understand and trust that their anonymity will be preserved and their personal information will be kept absolutely confidential. Situations of distrust about the seriousness and the purpose of the research generate fear and insecurity in the participants about how the information provided can be used. As a result, it is common for people to try to protect themselves by giving untrue answers.

### Characteristic of the Respondents

Some of the elements involving the characteristics of the respondents were previously addressed, when discussing self-deception and impression management. Another characteristic related to the respondents is the so-called demand effect. This situation happens when the respondent gives an answer that they believe will please the interviewer and when they try to give what they believe to be the expected answers^24^. This behavior is related to the acquiescence bias or the yea-saying bias, in which the respondent has a tendency to be positive and agree with everything the interviewer presents. This attitude is considered easier because it requires less effort than carefully thinking and elaborating each answer.

### The Interviewer's Position

Reporting socially reproved opinions and events to a person who does not inspire confidence is unlikely to happen. In this sense, the interviewer's characteristics, attitude and way of conducting the interview are strong determinants of the social desirability bias. Even the interviewer's personal characteristics, such as social class, ethnicity, gender, and personality traits can induce biased responses^1^. In addition, the researcher bears definitions, a specific language and a culture that dictates habits, ways of proceeding, preferences and norms to be followed^25^, which can influence the participants’ responses.

When it comes to people in communication, there is always a relational aspect, which is produced in the act of affecting and being affected by another person in the narrative mediation^26^. For that, it is essential to develop a relationship of trust between interviewer and interviewee. Aspects such as empathy, respect, good humor and warmth help the interviewee feel secure respond on sensitive topics.

Another important determinant concerns the interviewer's reactions to the answers given. The way the interviewer reacts to responses can encourage or inhibit certain positions. Graeff^12^ points out that a smile, a frowning countenance or even the raising of an eyebrow can indicate which answers the interviewer expects or disapproves. Consequently, respondents may censor or distort other positions.

The interviewer's skill is also critical to identifying biased responses and encouraging respondents to respond truthfully. Some behaviors of the interviewee may indicate the existence of bias, such as excessive discomfort, acquiescent responses and responses that contradict already identified evidence.

### Control Strategies and Interpretive Reflections of the Social Desirability Bias in Qualitative Health Research

Given the determining factors that influence the existence of social desirability bias, eight reflections aimed at identifying, reducing and interpreting this type of bias in qualitative health research were systematized.

Firstly, the planning phase of the research project should be carefully developed. Special attention and rigor should be given to the definition of objectives, the choice of research techniques, the selection of participants and the elaboration of the instruments. Situations of inadequate study design may imply systematic errors when obtaining information. Such situations can be irreversible and compromise the quality of the results achieved. Whenever possible, the researcher should choose more than one source of information in order to triangulate the data and identify socially desirable responses. In these cases, it is recommended that the interview or focus group take place after having access to information from other sources, such as participant observation and document analysis.

Secondly, special attention should be paid to the preparation of the interview script or focus group. Qualitative health research encompasses values, practices, beliefs, habits and attitudes of professional users and managers^8^, as objects often sensitive and difficult to approach. Thus, the questions should be formulated in such a way as to clarify that there is no problem in sharing positions or revealing socially disapproved actions. The order of the questions must also be noted^13^. It is recommended to start the interview with comprehensive questions rather than immediately asking questions about the topic of the research. This helps to break the initial tension and allows respondents to relax and gain confidence. When addressing specific content, it is suggested to start with more general questions about the content and then introduce sensitive subjects. In addition, words and expressions that are emotionally charged or imply value judgment about a particular behavior should be avoided.

Thirdly, ensuring privacy and a conducive atmosphere for the research context are key to reducing social desirability bias. The research environment must be protected from external influences, interruptions or the presence of third parties. In health studies, participants are often asked about highly sensitive topics, such as sexual practices, family relationships or situations of violence, which reinforces the need for a private environment for data collection. The researcher must have good judgment when chosing the spaces, ensuring that the participants are not heard by people in other environments. It is also necessary to properly prepare the space, with chairs, a table, water and other amenities that make the environment comfortable and help to avoid interruptions. Data should always be collected only in the presence of the research team and participants, with exceptions allowed to suit the respondent's needs, especially users.

Fourth, the confidentiality of data and information must always be safeguarded and users must be assured of their anonymity. Given the sensitivity of the topics of qualitative health research, personal identification of certain disclosures can generate moral, social, family, financial and legal losses. Therefore, it is necessary to assure respondents and make sure that they understand and trust that their anonymity will be preserved and their personal information will be kept absolutely confidential. Research involving human beings requires approval by a research ethics committee and compliance with the ethical aspects of the legislation in force. However, in many situations, the signing of the free and informed consent form and ethical and confidentiality clarifications occur as a mere procedural and bureaucratic step.

Fifth, unexpected participation in interviews and focus groups should be avoided. Respondents who are more familiar with their interviewers opt less for socially desirable responses^14^. That is, the continuity of the researcher in the field and the development of familiarity with the participants benefit the development of honest responses. When continuity in the field is not possible, it is recommended to contact the participants beforehand, when clarifications should be provided about the study, and to schedule participation for a later time.

Sixth, attention should be paid to the atitude and qualification of the researcher. Researchers should always seek to build a good relationship with participants and to promote a respectful and relaxed atmosphere. Scott et al.^27^ recommend using verbal and nonverbal language to make respondents feel comfortable and less hestitant to express unpleasant positions. When identifying socially desirable positions, it is important to avoid confrontation and use strategies to make the respondent understand the scientific nature of the research. As health research often deals with very specific topics that raise questions in the participants, it is essential to master the specific contents of the nature of the object. During the interview, doubts, questions and misunderstandings may arise that require specific knowledge from the researcher to provide proper clarification. Thus, proper cognitive and relational training of the researchers responsible for fieldwork is required.

Seventh, having the sensitivity to identify situations of desirability bias and reflect critically on the participants’ positions. Although instrumental and procedural resources should always be observed, it is not always possible to control the existence of bias, and in some situations, it is desirable not to do so. In certain contexts of qualitative health research, participants may deliberately distort situations experienced, such as political beliefs, when confronted by situations of oppression or in defense of certain cultural and community aspects, or even in actions aimed at transforming health services. In this sense, the existence of the social desirability bias takes on another perspective. The researcher is not expected to try to avoid it or control it. In these cases, the bias reveals important aspects to consider and analyze in depth, and the researcher must broaden the reflections and theorizations about the phenomenon under study and the revelations manifested through “biased opinions”.

Eighth, ethical-political and social attitude of the researcher in social sciences in health care. Social research, by nature, must be connected with everyday problems and committed to building a more just society. In this perspective, the recommendations to control the bias of social desirability are not only technical-procedural requirements, aimed at increasing the rigor in qualitative research, but also political stances with the goal of better understanding the world and finding ways to transform local and global realities.

## FINAL CONSIDERATIONS

This essay addressed the occurrence, determinants and strategies of approaches to the social desirability bias in qualitative health research. The existence of biases can compromise the consistency of the results of a scientific study and lead to conclusions that do not correspond exactly to the characteristics of the phenomenon being studied. In some situations, the existence of this type of bias may reveal situations of oppression and deliberate political stance experienced by the participants.

I highlight the importance of the attention and attitude of the researcher in social sciences and humanities in health in being alert to the possibility of the existence of this type of bias. Norms, customs, values and the social context exert a strong influence on the elaboration of responses by participants, and this cannot be ignored by researchers. The researcher must adopt strategies to minimize the occurrence of bias or, based on them, to interpret aspects of the participants’ experiences and meanings in depth.
