# Supplementary material for: Social desirability bias in qualitative health research
Source: Rev Saude Publica. 2022 Nov 18;56:101. doi: 10.11606/s1518-8787.2022056004164 (PMC9749714; doi:10.11606/s1518-8787.2022056004164)
Supplement: Translation

# Translation: Viés de desejabilidade social na pesquisa qualitativa em saúde

**Authors:** José Patrício Bispo

**Affiliations:** I Universidade Federal da Bahia Instituto Multidisciplinar em Saúde Vitória da Conquista BA Brasil Universidade Federal da Bahia. Instituto Multidisciplinar em Saúde. Vitória da Conquista, BA, Brasil

**Keywords:** Pesquisa Qualitativa, Viés, Desejabilidade Social, Metodologia, Pesquisa em Sistemas de Saúde Pública

## Abstract

Ensaio com o objetivo de discutir o viés de desejabilidade social na pesquisa qualitativa em saúde. O viés de desejabilidade social consiste em um erro sistemático de pesquisa, no qual o participante apresenta respostas que são mais socialmente aceitáveis do que suas opiniões ou comportamentos verdadeiros. Estudos qualitativos são muito suscetíveis a esse tipo de viés, que pode levar a conclusões distorcidas sobre o fenômeno em estudo. Inicialmente, apresento os aspectos teórico-conceituais do viés de desejabilidade social. Discuto como sua ocorrência pode ser intencional ou não intencional, com diferenciação entre os conceitos de autoengano e gerenciamento de impressão. Em seguida, discuto os fatores determinantes desse viés a partir de quatro dimensões: desenho do estudo; contexto do estudo; característica do entrevistado; postura do entrevistador. Por fim, apresento uma sistematização de seis estratégias a serem utilizadas por pesquisadores qualitativos para a identificação e controle do viés de desejabilidade social.

## INTRODUÇÃO

Vieses podem existir em pesquisas em saúde, tanto na pesquisa quantitativa quanto na pesquisa qualitativa^1^. Embora não seja um tema novo, a discussão sobre vieses na pesquisa qualitativa é ainda tímida e demanda a necessidade de maior atenção e aprofundamento por parte dos pesquisadores. Segundo Althubaiti^2^, o problema do viés é ainda frequentemente ignorado na prática. Para o autor, na maior parte dos casos, o viés é introduzido de maneira não intencional por pesquisadores em um estudo, situação que o torna difícil de ser reconhecido. Desse modo, constitui-se numa questão de grande relevância para o debate sobre o fortalecimento da consistência dos estudos qualitativos.

Viés de pesquisa pode ser definido como a influência de algum fator que provém distorções nos resultados do estudo^3^. Trata-se de erros sistemáticos que podem ocorrer em todas as etapas de desenvolvimento da pesquisa^4^: no planejamento e desenho do estudo; na coleta de dados; e na análise e interpretação das informações. Em qualquer uma das fases, a existência desse tipo de erro pode comprometer o rigor e a consistência dos achados da investigação.

Na pesquisa qualitativa, a discussão sobre a existência e o tratamento de vieses é controversa e não consensual. Características inerentes ao próprio método, como a obtenção de dados por interação verbal ou observação e a natureza interpretativa da análise, são frequentemente apontados como fontes incontroláveis de vieses que ameaçam a credibilidade da pesquisa^5^. Segundo Roulston e Shelton^6^, isso é devido à presunção dos valores positivistas e quantitativos sobre o método qualitativo.

Nesse debate, é importante delimitar os aspectos epistêmicos da pesquisa qualitativa em saúde. Segundo Guba e Lincoln^7^, a pesquisa qualitativa busca abranger a dimensão histórica, cultural e subjetiva dos fenômenos humanos. Portanto, a objetividade e neutralidade, tão reificadas nas ciências naturais, não se constituem em fundamento para as investigações qualitativas. A pesquisa qualitativa possui como referencial epistemológico o paradigma construtivista interpretativo em que a reflexividade e a subjetividade são valorizados como meios para interpretação de fenômenos sociais complexos^7,8^.

Trad^9^ ressalta que a vigilância epistêmica consiste em equilibrar a valorização da subjetividade com o imperativo de produzir conhecimento científico. Assim, é necessário se ater às contradições dos informantes e considerar as manipulações que possam tentar fazer. Diversas estratégias são utilizadas para tentar amenizar os fatores intervenientes que possam influenciar inadequadamente as realidades observadas ou os relatos dos participantes do estudo^10,11^.

A fase de coleta de dados é muito suscetível aos vieses de resposta. Por viés de resposta, entende-se a existência de erro de pesquisa resultante de afirmações deturpadas de maneira intencional ou não intencional^12^. Nessa situação, os entrevistados alteram, censuram ou deturpam suas verdadeiras opiniões, pensamentos e crenças. Como resultado, as respostas das questões não são representativas de como os participantes realmente se comportam, pensam ou sentem^12,13^.

Existem vários tipos de viés de resposta. Como exemplos, podem ser destacados^2,12,14^: o viés de memória; o viés de aquiescência, também conhecido como viés de dizer sim; viés de apatia ou viés de habituação; e o viés de desejabilidade social. Por viés de desejabilidade social entende-se a tendência de um participante do estudo em apresentar a si mesmo ou o seu contexto social de uma forma que seja socialmente aceitável, mas não totalmente correspondente à realidade^15^.

A existência desse tipo de viés é motivada pela predisposição das pessoas em negar traços socialmente indesejáveis e reivindicar outros socialmente desejáveis. Refere-se, assim, ao desejo de dizer coisas que causam uma boa impressão para pessoas com quem interagem em uma determinada situação^16^.

Diante de comportamentos socialmente reprováveis ou de violações de leis e normas, a obtenção de relatos sinceros e confiáveis constitui-se num grande desafio da pesquisa qualitativa. Conforme ressaltado, os estudos qualitativos em saúde são muito suscetíveis ao viés de desejabilidade social. No entanto, esse é um tema que tem recebido pouca atenção no debate sobre os aspectos metodológicos da pesquisa em saúde.

Este ensaio tem por objetivo discutir o viés de desejabilidade social na pesquisa qualitativa em saúde e apresentar potenciais estratégias de controle para esse tipo de viés. O texto está estruturado em três seções. Na primeira parte, apresento a discussão conceitual e as reflexões teórico-metodológicas sobre as respostas socialmente desejáveis. Na segunda seção, abordo os fatores determinantes desse tipo de viés, considerando as características de uma investigação qualitativa. Na terceira seção, apresento uma sistematização de algumas estratégias a serem utilizadas para identificação e controle do viés de desejabilidade social.

### Viés de Desejabilidade Social: Aspectos Conceituais e Metodológicos

O viés de desejabilidade social descreve um comportamento dos participantes de um estudo em fazer autodescrições positivas, a fim de criar imagens socialmente adequadas de si ou de determinadas situações em vez de responder de forma verdadeira e precisa^14^. Motivados por diversos fatores, os participantes tendem a superestimar comportamentos, atitudes e traços socialmente aceitáveis e subestimar opiniões e comportamentos verdadeiros, caso sejam socialmente indesejáveis^13,17^.

Respostas com revelações de desvios de normas sociais são vistas de maneira reprovável e, portanto, difícil de serem obtidas nas investigações científicas. Nesse sentido, os resultados obtidos pelos estudos podem não ser capazes de revelar muitos dos aspectos do objeto em investigação. Consequentemente, as conclusões apresentadas pelos autores podem estar distorcidas ou não expressar adequadamente o comportamento das pessoas, o funcionamento dos serviços de saúde ou o desenvolvimento das políticas públicas.

Relatos de comportamentos alinhados aos padrões socialmente estabelecidos são vistos como capazes de evitar reações de desprezo e muitas vezes associados a potenciais ganhos de uma boa imagem. Assim, os entrevistados podem distorcer suas respostas em direção à norma social a fim de manter uma autoapresentação socialmente favorável^17^.

O viés de desejabilidade social se relaciona com assuntos polêmicos ou comportamentos que fogem aos padrões legais, culturais e éticos estabelecidos em cada sociedade. Krumpal^17^ utiliza a denominação de temas sensíveis para expressar assuntos considerados tabu, revelam comportamentos ilegais ou expressam atitudes antissociais. Desse modo, obter informações confiáveis na investigação de temas sensíveis é um desafio para as ciências sociais em saúde. O autor apresenta três dimensões dos temas sensíveis: (1) intrusão, visto que certas questões podem ser percebidas como de natureza privada ou de fórum íntimo; (2) medo de divulgação, referente às preocupações dos entrevistados sobre potenciais riscos e consequências da divulgação das respostas para além do ambiente da pesquisa; e (3) desejabilidade social, referente à distorção das respostas em relação à norma social, a fim de manter uma autoapresentação socialmente favorável.

Uma questão importante desse debate é que o viés de desejabilidade social pode ser intencional ou não-intencional. De acordo com Paulhus^18^, respostas socialmente adequadas podem ser resultantes de duas situações: autoengano e gerenciamento de impressão. No autoengano, as distorções das respostas são motivadas por atributos de personalidade inflada e elevada autoestima^15^, o que favorece a tendência do entrevistado em se ver sempre de maneira positiva^12^. Assim, a pessoa realmente acredita que a afirmação sobre si é verdadeira, mesmo que a resposta seja imprecisa^15^. As respostas por autoengano são motivadas pela necessidade constante de aprovação social independentemente do que esteja sendo abordado^18^.

Por sua vez, o gerenciamento de impressão diz respeito à ação intencional de deturpar a verdade como forma de causar uma boa impressão^15^. Nessa situação, os entrevistados administram deliberada e conscientemente uma resposta a fim de se apresentar de maneira positiva, omitindo e deturpando fatos que possam gerar situações desfavoráveis^14^.

Distinguir essas duas perspectivas se mostra de grande relevância na pesquisa social em saúde, pois permite a separação dos determinantes passíveis de controle e interferência por parte do pesquisador. As situações de autoengano são menos facilmente controladas e podem, na maioria dos casos, apenas ser detectadas^16^. Por sua vez, o gerenciamento de impressão desenvolve-se a partir de uma situação ou de um item específico, o qual o participante do estudo busca esconder ou deturpar^17^. Como são influenciados pelas características de um item, são mais facilmente identificáveis e também possibilitam ao pesquisador desenvolver estratégias para prevenir ou contornar esse tipo de viés.

### Determinantes do Viés de Desejabilidade Social

A obtenção de informações na pesquisa qualitativa envolve os atores (entrevistador, entrevistados e potenciais espectadores), a relação estabelecida entre eles, os segmentos sociais ou as instituições às quais estão vinculados, o ambiente de realização do estudo e as normas socioculturais estabelecidas. Desse modo, algumas características do estudo, as circunstâncias de realização do mesmo e os posicionamentos dos atores envolvidos podem facilitar a ocorrência de vieses. Apresento a seguir uma sistematização dos fatores determinantes do viés de desejabilidade social.

### Desenho do Estudo

Antes da realização de qualquer estudo científico é necessária a cuidadosa atenção do pesquisador na definição dos objetivos propostos, dos métodos de pesquisa, das técnicas a serem utilizadas na obtenção de dados e da seleção dos participantes. Um importante passo para evitar o viés de desejabilidade social é analisar a pertinência e coerência entre os objetivos e os elementos metodológicos a serem seguidos.

A escolha da técnica de obtenção de dados pode facilitar ou dificultar respostas enviesadas. Duas técnicas de coleta na pesquisa qualitativa estão sujeitas ao viés de desejabilidade social: entrevista e grupo focal.

A entrevista é caracterizada como uma conversa com finalidade e constitui-se como a técnica mais utilizada no trabalho de campo qualitativo^19^. Vários fatores podem contribuir para os entrevistados não formularem as respostas com sinceridade, incluindo o desejo de omitir opiniões e comportamentos socialmente reprováveis, a vontade de demonstrar o domínio do conteúdo ou até mesmo querer agradar ao entrevistador. A natureza dinâmica da entrevista e a possibilidade do entrevistador identificar traços de desvios na fala do participante, permite o direcionamento e o uso de recursos para minimizar as abordagens enviesadas.

Por grupo focal, entende-se uma técnica de pesquisa qualitativa que coleta informações por meio das interações grupais^20^. O mesmo se baseia em gerar informações a partir da interação entre os participantes, ao invés de fazer a mesma pergunta de maneira individual^20,21^. Assim, um dos principais desafios do grupo focal é justamente promover a interação e o debate entre os participantes e fazer com que não interajam apenas com o moderador^21^. Frente às peculiaridades dos grupos focais, os mesmos podem se constituir em técnica capaz de amenizar ou potencializar o viés de desejabilidade social. Na interação entre os participantes, pode se desenvolver um processo de autocontrole do grupo, com capacidade para inibir opiniões e posicionamentos que não correspondam com a realidade em discussão. Por outro lado, é também possível que se desenvolva uma espécie de micropacto social para coletivamente esconder determinados comportamentos ou práticas que possam ser consideradas inadequadas.

Sobre os participantes do estudo, três grupos de pessoas são mais comumente requisitados na pesquisa qualitativa em saúde: usuários e cuidadores; profissionais de saúde; e gestores. O enviesamento das respostas dos participantes guarda forte relação com a sensibilidade dos temas abordados nos estudos e os critérios de seleção dos participantes. Por exemplo, usuários podem se sentir incomodados em revelar comportamentos sexuais de risco ou envergonhados de relatar situações de violência doméstica. A situação talvez seja ainda mais difícil para tratar com gestores de aspectos relacionados à forma de administração dos recursos ou, caso existam, sobre comportamentos ilegais, omissões e fraudes.

Com efeito, obter respostas sinceras e correspondentes com a realidade que se investiga não é uma tarefa fácil. Não raro, os participantes quando questionados sobre uma determinada prática ou o funcionamento de um determinado serviço adotam a postura de descrever uma situação ideal ou apresentar os parâmetros de uma dada política em vez de relatar a realidade cotidiana vivenciada por eles.

Os mecanismos de seleção dos participantes também podem influenciar na ocorrência do viés de desejabilidade social. Existem dois tipos básicos de definição dos participantes^22,23^: estabelecer a priori a quantidade e as características dos respondentes e selecionar conforme as necessidades e questões que aparecerem no decorrer da pesquisa. Em ambas as situações, selecionar adequadamente participantes imbricados e dispostos a revelar opiniões e comportamentos verdadeiros mostra-se um desafio para a garantia do rigor e para a diminuição da ocorrência de viés. A seleção inadequada pode repercutir nas etapas subsequentes do trabalho de campo e da análise de dados e obstaculizar ações para o controle do viés de desejabilidade social.

Outro importante aspecto relacionado ao desenho do estudo é a adequada elaboração dos instrumentos. A forma de redação do roteiro pode induzir o teor da resposta. De acordo com Kaminska e Foulsham^13^, a formulação da questão pode implicar que existe um comportamento ou atitude socialmente indesejável, levando as pessoas a responder de maneira tendenciosa. Ou seja, determinadas palavras ou frases contidas no instrumento sugerem determinados tipos de respostas^12^. Também a ordem das questões pode gerar vieses, uma vez que a resposta dada a uma questão pode influenciar nas respostas das questões subsequentes.

### Contexto do Estudo

Fatores contextuais da etapa de campo têm grande potencial para gerar vieses. Dois principais fatores contextuais influenciam a ocorrência do viés de desejabilidade social: o efeito espectador e a confidencialidade dos dados.

Por efeito espectador, entende-se a presença de uma ou mais pessoas, além do pesquisador e do participante, no momento da coleta de dados. Em face à elevada probabilidade de repercussões negativas, na presença de uma terceira parte, o entrevistado reportará menos respostas socialmente indesejáveis^17^. Nas pesquisas qualitativas em saúde, não é incomum pesquisadores se depararem com contextos que incluem uma terceira pessoa no momento da coleta. Por exemplo, entrevistas nos domicílios dos usuários quase sempre são acompanhadas de outros moradores, o que pode se constituir numa situação de difícil controle e afetar negativamente a qualidade das informações prestadas. O efeito espectador também pode ocorrer de maneira indireta, relacionado a falta de privacidade do ambiente da pesquisa. Em determinados ambientes, a exemplo de unidades de saúde ou espaços administrativos, em que as falas podem ser ouvidas por pessoas em outros espaços, existe uma maior propensão para a distorção das respostas.

Sobre a confidencialidade dos dados, é necessário assegurar e fazer com que o entrevistado compreenda e confie que terá o anonimato preservado e as suas informações pessoais mantidas em absoluto sigilo. Situações de desconfiança sobre a seriedade e finalidade da pesquisa geram receio e insegurança nos participantes sobre como as informações prestadas poderão ser utilizadas. Desse modo, é comum as pessoas buscarem se resguardar por meio de respostas não verdadeiras.

### Característica do Entrevistado

Alguns dos elementos que envolvem as características do entrevistado foram anteriormente abordados, quando discutidos o autoengano e o gerenciamento de impressão. Outra característica relativa ao entrevistado é o chamado efeito de demanda. Essa situação se caracteriza por uma resposta do entrevistado visando agradar ao entrevistador e buscar as respostas que acredita serem as esperadas^24^. Tal postura se relaciona com o viés de aquiescência ou viés de dizer sim, em que o entrevistado apresenta uma tendência de ser positivo e concordar com tudo o que o entrevistador apresentar. Trata-se de um posicionamento considerado mais fácil, pois exige menos esforço do que pensar e elaborar cuidadosamente cada resposta.

### Postura do Entrevistador

Relatar opiniões e acontecimentos socialmente reprováveis para uma pessoa que não inspira confiança é algo pouco provável de acontecer. Nesse sentido, as características, posturas e forma de condução do entrevistador são fortes determinantes do viés de desejabilidade social. As próprias características individuais do entrevistador, como posição social, etnia, gênero e traços de personalidade podem induzir a respostas enviesadas^1^. Além do mais, o pesquisador é um ser portador de definições, de uma linguagem própria e de uma cultura que lhe dita hábitos, maneiras de proceder, preferências e normas a seguir^25^, o que pode influenciar nas respostas dos participantes.

Quando se trata de sujeitos em comunicação, há sempre um aspecto relacional, que é produzido na ação de afetar e ser afetado por outra pessoa na mediação narrativa^26^. Para tanto, é fundamental o desenvolvimento de uma relação de confiança entre entrevistador e entrevistado. Aspectos como empatia, respeito, bom humor e cordialidade favorecem a segurança do entrevistado para responder sobre temas sensíveis.

Outro importante determinante diz respeito às reações do entrevistador ante as respostas proferidas. A forma como o entrevistador reage às respostas pode encorajar ou inibir determinados posicionamentos. Graeff^12^ ressalta que um sorriso, um semblante carrancudo ou até mesmo o levantar de uma sobrancelha podem indicar quais as respostas esperadas ou reprovadas pelo entrevistador. Consequentemente, os entrevistados podem censurar ou distorcer outros posicionamentos.

A habilidade do entrevistador também é fundamental para identificar respostas enviesadas e encorajar os entrevistados a responderem com sinceridade. Alguns comportamentos do entrevistado podem indicar a existência de viés a exemplo de desconforto excessivo, respostas aquiescentes e respostas que contrariam evidências já identificadas.

### Estratégias de Controle e Reflexões Interpretativas do Viés de Desejabilidade Social na Pesquisa Qualitativa em Saúde

Diante dos fatores determinantes e que influenciam na existência do viés de desejabilidade social, foram sistematizadas oito reflexões destinadas à identificação, redução e interpretação desse tipo de viés na pesquisa qualitativa em saúde.

Primeira, a fase de planejamento do projeto de pesquisa deve ser cuidadosamente desenvolvida. Deve-se destinar especial atenção e rigor para a definição dos objetivos, a escolha das técnicas de investigação, a seleção dos participantes e a elaboração dos instrumentos. Situações de desenho inadequado do estudo podem implicar em erro sistemático de obtenção das informações. Tais situações podem ser irreversíveis e comprometer a qualidade dos resultados alcançados. Sempre que possível, o pesquisador deve optar por mais de uma fonte de informação a fim de triangular os dados e identificar respostas socialmente desejáveis. Nesses casos, é recomendado que a entrevista ou grupo focal ocorra após se ter acesso às informações das outras fontes, a exemplo da observação participante e da análise de documentos.

Segunda, deve-se dedicar especial atenção à elaboração do roteiro da entrevista ou grupo focal. A pesquisa qualitativa em saúde engloba valores, práticas, crenças, hábitos e atitudes de usuários profissionais e gestores^8^, como objetos muitas vezes sensíveis e de difícil abordagem. Assim, as perguntas devem ser formuladas de modo a esclarecer que não há problema em apresentar posicionamentos ou revelar ações socialmente reprováveis. Também deve ser observado a ordem das questões^13^. No início da entrevista, é recomendado realizar perguntas abrangentes e não questionar de imediato o tema da pesquisa. Isso ajuda a quebrar a tensão inicial e possibilita o relaxamento e ganho de confiança dos entrevistados. Ao abordar os conteúdos específicos, sugere-se iniciar com perguntas mais gerais sobre o conteúdo e em seguida introduzir os assuntos sensíveis. Além do mais, devem ser evitadas palavras e expressões carregadas de emoção ou que impliquem juízo de valor sobre determinado comportamento.

Terceira, a garantia da privacidade e de um clima adequado ao contexto da pesquisa são fundamentais para reduzir o viés de desejabilidade social. O ambiente da pesquisa deve estar resguardado de influências externas, interrupções ou presença de terceiros. Com frequência, nas investigações em saúde os participantes são questionados sobre temas de grande sensibilidade, como a respeito de práticas sexuais, relações familiares ou situações de violência, o que reforça a necessidade de ambiente privativo para coleta de dados. É necessário que o pesquisador seja criterioso na escolha dos espaços, assegurando que os participantes não sejam ouvidos por pessoas em outros ambientes. É necessário também preparar adequadamente o espaço, com cadeiras, mesa, água e outras comodidades que tornem o ambiente confortável e ajude a evitar interrupções e quebras de raciocínios. Os dados devem ser sempre coletados apenas na presença da equipe de pesquisa e dos participantes, com exceções apenas para situações de necessidade do entrevistado, especialmente usuários.

Quarta, a confidencialidade dos dados e informações devem ser sempre resguardadas e os usuários devem estar convencidos da garantia do anonimato. Frente à sensibilidade dos assuntos da pesquisa qualitativa em saúde, a identificação pessoal de determinadas revelações pode gerar prejuízos morais, sociais, familiares, financeiros e legais. Assim, é necessário assegurar e fazer com que o entrevistado compreenda e confie que terá o anonimato preservado e as suas informações pessoais mantidas em absoluto sigilo. Pesquisas envolvendo seres humanos necessitam da aprovação em comitê de ética em pesquisa e da observância dos aspectos éticos da legislação em vigor. No entanto, em muitas situações, a assinatura do Termo de Consentimento Livre e Esclarecido e os esclarecimentos éticos e sobre a confidencialidade ocorre apenas como etapa procedimental e burocrática.

Quinta, deve ser evitada a participação repentina nas entrevistas e grupos focais. Entrevistados que estão mais familiarizados com os entrevistadores optam menos por respostas socialmente desejáveis^14^. Ou seja, a continuidade do pesquisador em campo e o desenvolvimento da familiaridade com os participantes favorecem o desenvolvimento de respostas sinceras. Quando a continuidade no campo não é possível, é recomendado o contato prévio com os participantes, quando se deve prestar esclarecimentos sobre o estudo e agendar a participação para um momento posterior.

Sexta, deve-se estar atento à postura e qualificação do pesquisador. Os pesquisadores devem sempre buscar desenvolver uma boa relação com os participantes e promover um clima respeitoso e descontraído. Scott et al.^27^ recomendam fazer o uso de linguagens verbais e não verbais para deixar os entrevistados à vontade e menos desconfortáveis para expressar posicionamentos desagradáveis. Ao identificar posicionamentos socialmente desejáveis, é importante evitar o confronto e buscar estratégias para fazer o entrevistado compreender o caráter científico da pesquisa. Como a pesquisa em saúde muitas vezes trata de temas muitos específicos que suscitam dúvidas dos participantes é imprescindível o domínio sobre os conteúdos específicos da natureza do objeto. Durante a entrevista podem surgir dúvidas, questionamentos e incompreensões que demandam do pesquisador conhecimentos específicos para prestar os devidos esclarecimentos. Assim, é necessária a adequada formação cognitiva e relacional dos pesquisadores responsáveis pelo trabalho de campo.

Sétima, ter a sensibilidade de identificar situações de viés de desejabilidade e refletir de maneira crítica sobre os posicionamentos dos participantes. Embora recursos instrumentais e procedimentais devam sempre ser observados, nem sempre é possível controlar a existência do viés e em algumas situações é desejável que não se faça. Em determinados contextos da pesquisa qualitativa em saúde, os participantes podem deliberadamente distorcer situações vivenciadas, como posicionamento político, frente a situações de opressão ou em defesa de determinados aspectos culturais e comunitários ou ainda em ação destinada à transformação dos serviços de saúde. Nesse sentido, a existência do viés de desejabilidade social assume outra perspectiva. Não cabe ao pesquisador buscar evitá-lo ou controlá-lo. Nesses casos, o viés é revelador de aspectos importantes a se considerar e aprofundar, cabendo ao pesquisador ampliar as reflexões e teorizações sobre o fenômeno em estudo e as revelações manifestadas por meio de “opiniões enviesadas”.

Oitava, postura ético-política e social do pesquisador em ciências sociais em saúde. A pesquisa social, por natureza, deve ser conectada com os problemas cotidianos e comprometida com a construção de uma sociedade mais justa. Nessa perspectiva, as recomendações para controlar o viés de desejabilidade social não são apenas requisitos técnico-procedimentais, destinados a ampliar o rigor na pesquisa qualitativa, são, antes, posicionamentos políticos com a finalidade de melhor compreensão do mundo e de buscar caminhos para a transformação das realidades locais e global.

## CONSIDERAÇÕES FINAIS

O ensaio abordou a ocorrência, os determinantes e as estratégias de abordagens frente ao viés de desejabilidade social na pesquisa qualitativa em saúde. A existência de vieses pode comprometer a consistência dos resultados de uma investigação científica e levar a conclusões que não correspondem com exatidão às características do fenômeno em estudo. Em algumas situações a existência desse tipo de viés pode ser reveladora de situações de opressão e de posicionamento político deliberado dos participantes.

Destaco a importância da atenção e postura do pesquisador em ciências sociais e humanas em saúde de estar em alerta para a possibilidade de existência desse tipo de viés. Normas, costumes, valores e o contexto social exercem forte influência na elaboração das respostas pelos participantes e isso não pode ser ignorado pelos pesquisadores. Cabe ao pesquisador adotar estratégias para minimizar a ocorrência do viés ou, a partir deles, interpretar e aprofundar aspectos das vivências e significados dos participantes.
